# Macrophages Compensate for Loss of Protein Tyrosine Phosphatase N_2_ in Dendritic Cells to Protect from Elevated Colitis

**DOI:** 10.3390/ijms22136820

**Published:** 2021-06-25

**Authors:** Larissa Hering, Egle Katkeviciute, Marlene Schwarzfischer, Anna Niechcial, Julianne B. Riggs, Marcin Wawrzyniak, Kirstin Atrott, Marnix van de Sande, Silvia Lang, Burkhard Becher, Gerhard Rogler, Michael Scharl, Marianne R. Spalinger

**Affiliations:** 1 Department of Gastroenterology and Hepatology, University Hospital Zurich, University of Zurich, Rämistrasse 100, 8091 Zurich, Switzerland; larissa.hering@usz.ch (L.H.); Egle.Katkeviciute@usz.ch (E.K.); Marlene.Schwarzfischer@usz.ch (M.S.); Anna.Niechcial@usz.ch (A.N.); jriggs@wesleyan.edu (J.B.R.); Marcin.Wawrzyniak@usz.ch (M.W.); Kirstin.Atrott@usz.ch (K.A.); m.a.j.vandesande@students.uu.nl (M.v.d.S.); Silvia.Lang@usz.ch (S.L.); Gerhard.Rogler@usz.ch (G.R.); Marianne.Spalinger@usz.ch (M.R.S.); 2 Institute of Experimental Immunology, University of Zurich, 8091 Zurich, Switzerland; becher@immunology.uzh.ch

**Keywords:** PTPN2, dendritic cells, inflammatory bowel disease, colitis

## Abstract

Protein tyrosine phosphatase nonreceptor type 2 (PTPN2) plays a critical role in the pathogenesis of inflammatory bowel diseases (IBD). Mice lacking PTPN2 in dendritic cells (DCs) develop skin and liver inflammation by the age of 22 weeks due to a generalized loss of tolerance leading to uncontrolled immune responses. The effect of DC-specific PTPN2 loss on intestinal health, however, is unknown. The aim of this study was to investigate the DC-specific role of PTPN2 in the intestine during colitis development. PTPN2^fl/fl^xCD11c^Cre^ mice were subjected to acute and chronic DSS colitis as well as T cell transfer colitis. Lamina propria immune cell populations were analyzed using flow cytometry. DC-specific PTPN2 deletion promoted infiltration of B and T lymphocytes, macrophages, and DCs into the lamina propria of unchallenged mice and elevated Th1 abundance during acute DSS colitis, suggesting an important role for PTPN2 in DCs in maintaining intestinal immune cell homeostasis. Surprisingly, those immune cell alterations did not translate into increased colitis susceptibility in acute and chronic DSS-induced colitis or T cell transfer colitis models. However, macrophage depletion by clodronate caused enhanced colitis severity in mice with a DC-specific loss of PTPN2. Loss of PTPN2 in DCs affects the composition of lamina propria lymphocytes, resulting in increased infiltration of innate and adaptive immune cells. However, this did not result in an elevated colitis phenotype, likely because increased infiltration of macrophages in the intestine upon loss of PTPN2 loss in DCs can compensate for the inflammatory effect of PTPN2-deficient DCs.

## 1. Introduction

Current hypotheses suggest that genetic and environmental factors contribute to the pathology of inflammatory bowel disease (IBD) by driving uncontrolled and excessive immune responses against the commensal intestinal microbiota [1]. Genome-wide association studies identified variants in the gene locus encoding protein tyrosine phosphatase nonreceptor type 2 (PTPN2; also known as T cell protein tyrosine phosphatase (TCPTP)) to be associated with IBD, as well as with other chronic inflammatory diseases, including type 1-diabetes, rheumatoid arthritis, and celiac disease [2,3,4]. First insights into the mechanisms by which PTPN2 variants might promote susceptibility to inflammatory disorders came from the observation that PTPN2 negatively regulates proinflammatory signaling cascades and that presence of the disease-associated variants results in a loss of function PTPN2 protein [5,6,7,8]. Well-described PTPN2 targets include signaling transducer and activator of transcription (STAT) molecules, mitogen-activated protein kinase (MAPK) P38, c-Jun N-terminal kinase (JNK), and extracellular signal-regulated kinase (ERK) [9,10,11,12], explaining the susceptibility to inflammatory disorders upon loss of functional PTPN2.

PTPN2-deficient (Ptpn2^−/−^) mice display severe immune defects with multiple organ dysfunction as well as intestinal and systemic inflammation, which is lethal within few weeks after birth [13,14]. PTPN2 is ubiquitously expressed [15], but the contribution of specific cell types to the systemic inflammatory phenotype in full-body Ptpn2^−/−^ mice is only partly understood. Loss of PTPN2 in T cells increases the number of effector and memory CD8^+^ T cells, affects the differentiation of CD4^+^ T cells, and results in increased susceptibility to intestinal inflammation [16,17]. In intestinal epithelial cells, loss of PTPN2 promotes inflammatory cytokine secretion and compromises barrier function in vitro but has no overt effect on intestinal inflammation in vivo during experimental DSS-induced colitis [7,18]. In contrast, loss of PTPN2 in myeloid cells promotes intestinal inflammation while protecting from colitis-associated tumor formation via increased inflammasome assembly and subsequent IL-1β production/maturation [19]. Further, loss of PTPN2 in dendritic cells (DCs) promotes DC activation, subsequently triggering aberrant T cell activation, which culminates in spontaneous inflammation of skin and liver in older mice [20].

Intestinal DCs have been shown to display distinct functions by balancing immune tolerance to harmless commensal bacteria and food antigens while mounting protective immune responses against invading pathogens [21,22]. Evidence suggests that colonic lamina propria DCs exert immune-regulatory functions in the intestine by promoting intestinal homeostasis and recruiting phagocytic cells to the site of inflammation [23,24]. As such, DC activation markers are upregulated in murine models of colitis, as well as in human CD and UC patients [25,26,27]. Given the crucial role of DC-intrinsic PTPN2 for sustained tissue tolerance and suppression of immune activation in skin and liver [20], we hypothesized that PTPN2 in DCs might also play a central role in preventing overshooting inflammatory responses in the intestine.

Our experiments demonstrate that DC-intrinsic PTPN2 controls and regulates intestinal immune cell composition. However, in the setting of colonic inflammation, loss of PTPN2 in DCs is compensated by other phagocytic cells. 

## 2. Results

### 2.1. Loss of PTPN2 in DCs Affects Immune Cell Infiltrations in Lamina Propria

**Figure 1:**
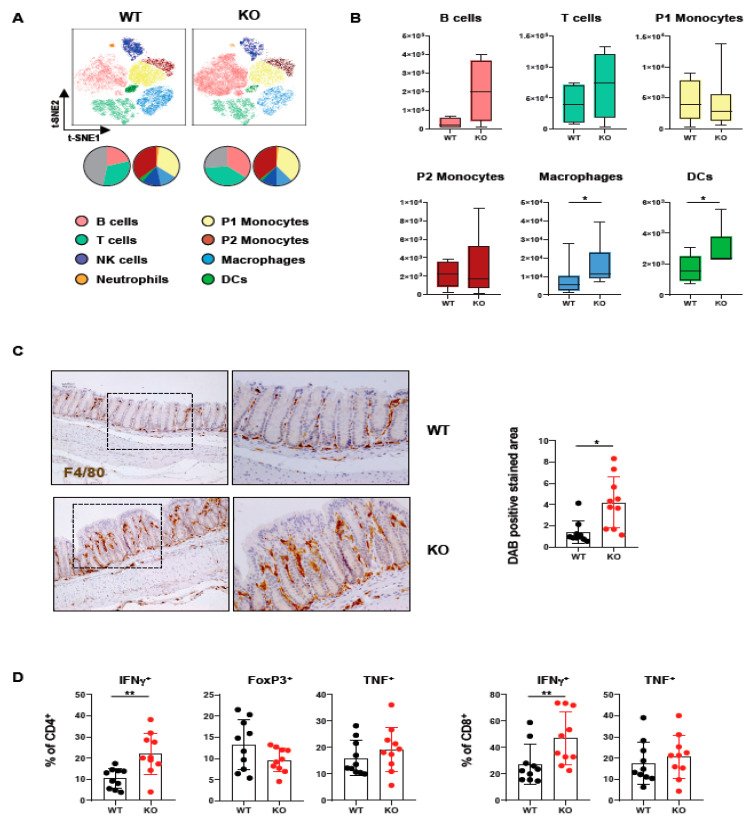
Loss of PTPN2 in DCs affects immune cell populations in the lamina propria. Immune cells were analyzed in PTPN2^fl/fl^ (WT) and PTPN2^fl/fl^xCD11c^Cre^ (KO) mice: (**A**,**B**) t-SNE maps displaying live, CD45^+^ singlet cells in lamina propria lymphocytes (LPL). Colors correspond to FlowSOM-guided clustering of cell populations. Pie charts represent relative numbers among CD45^+^ cells; (**A**) t-SNE maps of CD45^+^ cells in LPL; (**B**) total counts of indicated cell populations in LPL; (**C**) IHC staining for F4/80^+^ macrophages in colon; (**D**) relative numbers of Tregs, IFNγ- or TNF-producing CD4^+^ and CD8^+^ T cells. * *p* < 0.05, ** *p* < 0.01; unpaired Mann–Whitney test, *n* = 5–7 per group.

In order to assess how loss of PTPN2 in DCs affects immune cells in the colon, we analyzed lamina propria mononuclear cells of mice lacking PTPN2 specifically in DCs (PTPN2^fl/fl^xCD11c^Cre^ mice). Meta-clustering provided a comprehensive overview of the different immune cell populations (Figure 1A). Data was visualized in a t-distributed stochastic neighbor embedding (t-SNE) map and cells categorized using FlowSOM algorithm-guided clustering [28]. Traditional manual gating confirmed the identified cell populations (Supplementary Figure S1A). We identified changes in various immune cell populations, including B cells, T cells, and innate immune cells (Figure 1A). Overall, the number of infiltrating macrophages and DCs was increased, while B and T cells only showed a trend toward elevated numbers in knock-out mice, albeit this was not statistically significant. In contrast, P1 and P2 monocytes revealed comparable levels in PTPN2^fl/fl^xCD11c^Cre^ mice (Figure 1B). The increase of macrophages in the colon was confirmed by IHC staining for F4/80^+^ cells (Figure 1C). Given the moderate increase in T cells, we investigated whether loss of PTPN2 in DCs affects T cell subsets and cytokine-producing T cell populations (Supplementary Figure S1B). Here, we observed an increase in IFNγ-producing CD4^+^ and CD8^+^ T cells in PTPN2^fl/fl^xCD11c^Cre^ mice (Figure 1D). Together, these data show that loss of PTPN2 in DCs has a profound effect on innate and adaptive immune cells in the colonic lamina propria, pointing toward a generally elevated proinflammatory state in the colon of unchallenged PTPN2-deficient animals.

### 2.2. PTPN2 Deficiency in DCs Has No Impact on Intestinal Inflammation in Acute DSS Colitis

**Figure 2:**
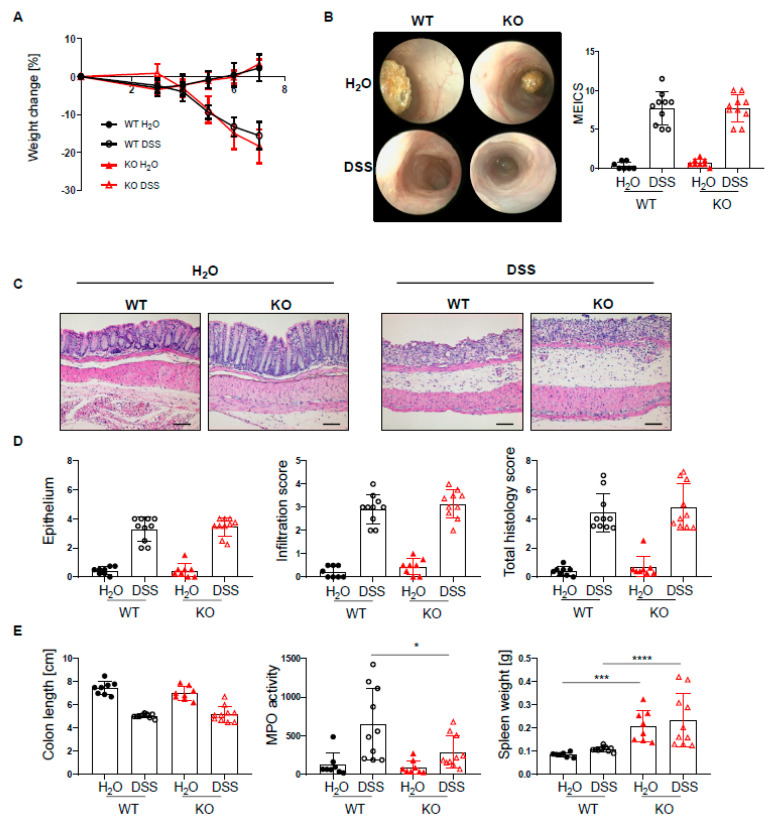
PTPN2 deficiency in DCs does not aggravate inflammation in acute DSS colitis. PTPN2^fl/fl^ (WT) and PTPN2^fl/fl^xCD11c^Cre^ (KO) mice received 2.5% DSS for 7 days: (**A**) relative weight changes; (**B**) representative pictures from colonoscopy and murine endoscopic index of colitis severity (MEICS); (**C**,**D**) representative pictures from H&E staining of the terminal colon and histological scoring; (**E**) colon length, MPO activity, and spleen weight. Scale bars represent a length of 100 mm. * *p* < 0.05, *** *p* < 0.001, **** *p* < 0.0001; unpaired Mann–Whitney test. Data are representative for one out of two independent experiments with 4–5 mice per experimental group.

Given the increased infiltration of innate and adaptive immune cells into the lamina propria of PTPN2^fl/fl^xCD11c^Cre^ mice, we next set out to investigate how the loss of PTPN2 in DCs might affect inflammation during colitis induction. To induce acute colitis, mice received 2.5% DSS in the drinking water for 7 days, which induced a similar and severe weight loss in both genotypes (Figure 2A). Colonoscopy revealed no difference in the MEICS score between PTPN2-deficient and control animals at the end of the experiment (Figure 2B). While histological scores were clearly increased in DSS-treated animals, no effect of PTPN2-deficiency in DCs on colitis severity could be seen (Figure 2C,D). Colitis induction led to a shortening of the colon in DSS-treated animals but DC-specific PTPN2-deficiency had no impact (Figure 2E). However, DSS-induced myeloperoxidase activity, a measure for active granulocytes, was lower in PTPN2^fl/fl^xCD11c^Cre^ mice (Figure 2E), whereas the spleen weight, a measure for systemic inflammatory responses, was increased in KO mice in both DSS and H_2_O groups (Figure 2E). Together, these data indicate that PTPN2 in DCs does not affect acute DSS-induced colonic inflammation.

### 2.3. Increased Th1 Response in PTPN2^fl/fl^xCD11c^Cre^ Mice in Acute Colitis

**Figure 3:**
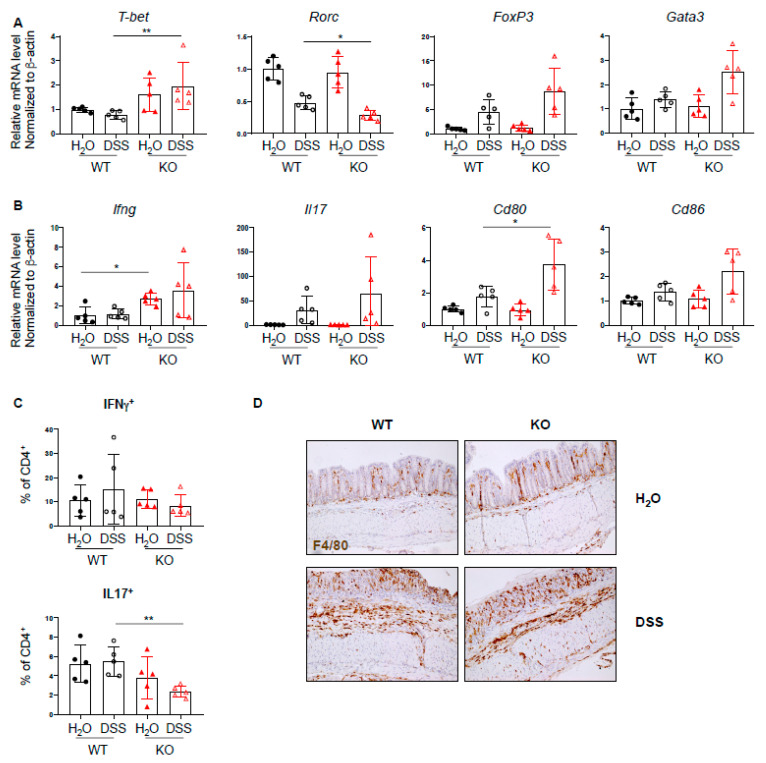
Increased Th1 response in PTPN2^fl/fl^xCD11c^Cre^ mice in acute colitis. RNA was isolated from whole colon pieces of PTPN2^fl/fl^ (WT) and PTPN2^fl/fl^xCD11c^Cre^ (KO) mice: (**A**,**B**) mRNA expression of the indicated; (**A**) Th-cell-associated transcription factors; (**B**) cytokines and activation markers in whole colon lysates; (**C**) frequencies of IFNγ^+^ and IL17^+^ CD4^+^ T cells in lamina propria; (**D**) IHC staining for F4/80^+^ macrophages in the colon. * *p* < 0.05, ** *p* < 0.01; unpaired Mann–Whitney test. Data are representative for one out of two independent experiments with 4–5 mice per experimental group.

Since PTPN2 has an inhibitory effect on several proinflammatory signaling cascades and plays a role in T cell development, we investigated whether loss of PTPN2 in DCs results in a change in T cell-associated cytokines and transcription factors during DSS-induced colitis. mRNA expression levels of several inflammation-related cytokines, transcription factors, and co-stimulatory molecules were analyzed by quantitative PCR. We found increased mRNA levels of the Th1-associated transcription factor *Tbet*, as well as a trend toward increased levels of Treg- and Th2-associated transcription factors *Foxp3* and *Gata3*, respectively, and reduced levels of Th17-associated *Rorc* (Figure 3A). Further, the mRNA level of *Ifng* was elevated in PTPN2^fl/fl^xCD11c^Cre^ mice irrespective of treatment, *Il17* was increased in DSS-treated groups and *Cd80* and *Cd86* were increased in DSS-treated PTPN2-deficient animals (Figure 3B). An increase in mRNA levels of *Tnfa*, *Tcrb, Il6*, and *Il10* was observed in both DSS-treated groups, although this was not significant due to large inter-individual variation (Supplementary Figure S2A). Given the increased expression of *Tbet* but reduced levels of *Rorc*, we investigated infiltrating Th-populations in the lamina propria and observed a decrease of IL17^+^CD4^+^ T cells in DSS-treated PTPN2^fl/fl^xCD11c^Cre^ mice (Figure 3C). Consistent with our findings in unchallenged mice, we again detected increased proportions of F4/80^+^ macrophages in untreated PTPN2^fl/fl^xCD11c^Cre^ mice. Upon DSS treatment, however, the observed infiltration of F4/80^+^ macrophages was similar to WT mice (Figure 3D). Together, this indicates that under inflammatory conditions, lack of PTPN2 in DCs promotes the expression of co-stimulatory molecules and affects Th-cell composition; however, this does not appear to provoke elevated colitis severity.

### 2.4. DC-Specific PTPN2 Regulates Phosphorylation of STAT1 in Acute DSS Colitis

**Figure 4:**
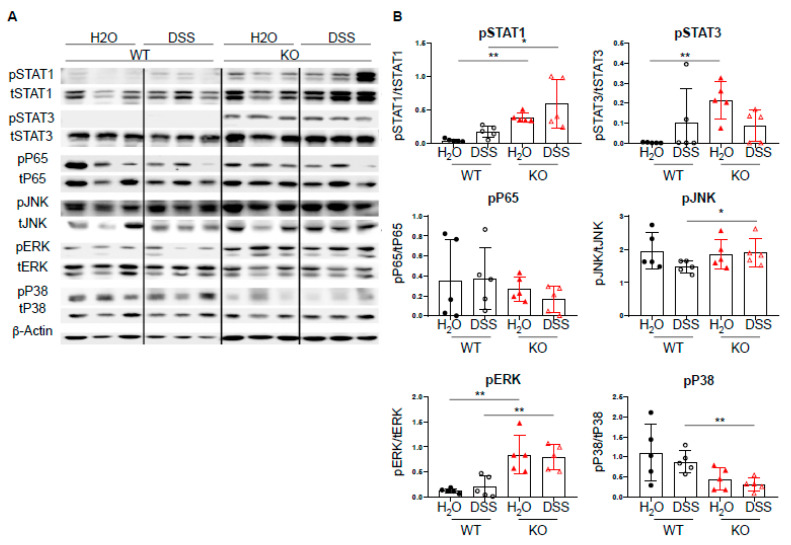
DC-specific PTPN2 regulates phosphorylation of STAT1 in acute DSS colitis. Protein lysates from whole colon pieces of PTPN2^fl/fl^ (WT) and PTPN2^fl/fl^xCD11c^Cre^ (KO) mice were analyzed by Western blot for the indicated proteins: (**A**) pictures show representative blots from 3 mice per group; (**B**) densitometric analysis, phosphorylated proteins are normalized to their respective total forms. * *p* < 0.05, ** *p* < 0.01; unpaired Mann–Whitney test. Data are representative for one out of two independent experiments with 4–5 mice per experimental group.

Given the increased expression of Th1-associated transcription factors, we next investigated the effect of DC-specific PTPN2 on well-described PTPN2 targets, such as STAT molecules, MAPK, JNK, and ERK [6,9,10,11]. Upon acute DSS-induced colitis, we observed increased levels of pSTAT1 in PTPN2^fl/fl^xCD11c^Cre^ mice upon DSS treatment, whereas pP65 and pJNK levels were not affected. PTPN2-deficient mice displayed increased levels of pERK and decreased levels of pP38, respectively. Further, we detected elevated levels of pSTAT3 in untreated PTPN2^fl/fl^xCD11c^Cre^ mice but upon DSS treatment, this difference was no longer present (Figure 4A,B). Taken together, despite the well-described action of PTPN2 on multiple signaling cascades, loss of DC-specific PTPN2 resulted in increased levels of STAT1 phosphorylation in acute DSS-induced colitis but had no drastic changes in the overall inflammatory response.

### 2.5. No Changes in PTPN2^fl/fl^xCD11c^Cre^ Mice upon Induction of Chronic Inflammation

**Figure 5:**
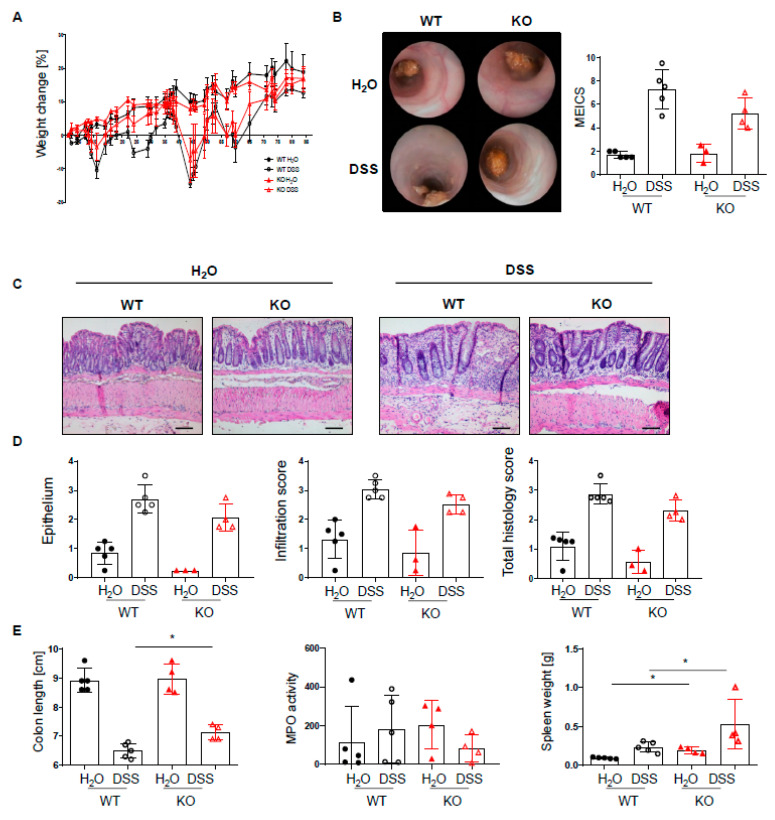
PTPN2^fl/fl^xCD11c^Cre^ mice tend to develop a less pronounced chronic inflammation. PTPN2^fl/fl^ (WT) and PTPN2^fl/fl^xCD11c^Cre^ (KO) mice received 4 cycles of DSS (7 days of 1.5% DSS followed by 10 days with normal drinking water) and were analyzed 4 weeks after the last DSS administration: (**A**) relative weight changes; (**B**) representative pictures from colonoscopy and assessment of murine endoscopic index of colitis severity (MEICS); (**C**,**D**) representative pictures from H&E staining of the terminal colon and histological scoring; (**E**) colon length, spleen weight, and MPO activity. Scale bars represent a length of 100 mm. * *p* < 0.05; unpaired Mann–Whitney test. Data are representative for one out of two independent experiments with 5 mice per experimental group.

Dendritic cells are important mediators between innate and adaptive immunity. However, in the short course of acute DSS colitis, adaptive immunity plays a minor role. To investigate whether prolonged inflammation and thus the involvement of the adaptive immune system would reveal an effect of PTPN2-deficient DCs on disease outcome, PTPN2^fl/fl^xCD11c^Cre^ mice were subjected to chronic DSS-induced colitis by administration of 1.5% DSS for four cycles, followed by a final recovery phase of 4 weeks. While by the end of the experiment, there was no difference in weight loss between genotypes (Figure 5A), in the acute phase of each cycle, the weight loss of PTPN2^fl/fl^xCD11c^Cre^ mice was less pronounced when compared to WT mice. This trend was also observed in terms of other colitis parameters, and PTPN2^fl/fl^xCD11c^Cre^ animals revealed a trend toward reduced colonoscopy and histological colitis scores (Figure 5B–D). Loss of PTPN2 resulted in less pronounced colon shortening, while myeloperoxidase activity was not altered and spleen weight was increased in PTPN2-deficient animals irrespective of treatment (Figure 5E). Similar to the observations in acute DSS colitis, we observed increased mRNA levels of Th1-associated *Tbet* (Supplementary Figure S2B,C). However, levels of *Foxp3*, inflammation-induced cytokines, and co-stimulatory molecules were comparable to WT animals or even slightly decreased (Supplementary Figure S2B,C), which is consistent with the slightly decreased colitis severity. Further, in contrast to our observations in acute DSS, DC-specific PTPN2 loss did not increase STAT1 phosphorylation nor affect other targets (Supplementary Figure S3A,B). Taken together, this indicates that—in contrast to our hypothesis—loss of PTPN2 in DCs clearly does not promote susceptibility to chronic DSS-induced colitis.

### 2.6. Loss of PTPN2 in DCs Has No Effect on Colitis Severity in a T Cell Transfer Colitis Model

**Figure 6:**
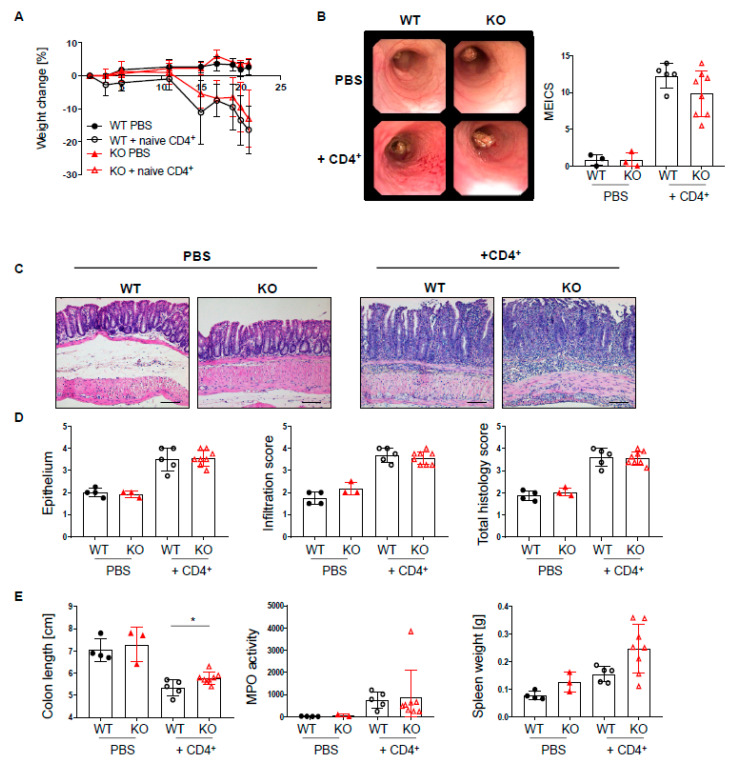
Loss of PTPN2 in DCs does not aggravate colitis severity in a T cell transfer colitis model. PTPN2^fl/fl^xRAG^−/−^ (WT) and PTPN2^fl/fl^xCD11c^Cre^xRAG^−/−^ (KO) mice were injected i.p. with PBS or 2.5 × 10^5^ naive CD4^+^ T cells: (**A**) weight development post injection; (**B**) representative pictures from colonoscopy and assessment of colitis severity according to the murine endoscopic index of colitis severity (MEICS); (**C**,**D**) representative pictures from H&E staining of the terminal colon and histological scoring; (**E**) colon length, spleen weight, and MPO activity. Scale bars represent a length of 100 mm. * *p* < 0.05; unpaired Mann–Whitney test. Data are representative for one out of two independent experiments with 3–5 mice per experimental group.

Given the profound changes in T cell subsets in the lamina propria of PTPN2^fl/fl^xCD11c^Cre^ mice, we further characterized the effect of PTPN2-deficiency in DCs in a T cell-driven colitis model. Naïve T cells were injected into PTPN2^fl/fl^xRAG2^−/−^ (WT) and PTPN2^fl/fl^xCD11c^Cre^xRAG2^−/−^ (KO) hosts. T cell injection induced weight loss from day 10 onwards in both WT and KO hosts, and weight loss in WT and KO mice was almost the same by the end of the experiment (Figure 6A). Colonoscopy at the end of the experiment revealed severe inflammation in the groups that received naïve T cells, and although there was a trend toward reduced colitis severity in KO mice, the difference was not significant (Figure 6B). Moreover, histological assessment of the colon revealed severe inflammation in the groups that received naïve T cells, but there were no genotype-dependent differences (Figure 6C,D). Further, PTPN2-deficient mice displayed less pronounced colon shortening while myeloperoxidase activity and spleen weight was increased in both groups that received naïve T cells (Figure 6E). In contrast to acute and chronic DSS colitis, T cell-associated transcription factors or inflammatory cytokines were not altered, but we detected slightly increased mRNA levels of *Il6*, *CD80*, and *Cd86* in PTPN2^fl/fl^xCD11c^Cre^xRAG^−/−^ mice after naïve T cell transfer (Supplementary Figure S4A,B). Additionally, flow cytometry analysis of lymphocytes isolated from colonic lamina propria revealed increased infiltration of IFN-γ^+^CD4^+^ T cells in PTPN2^fl/fl^xCD11c^Cre^xRAG^−/−^ mice after naïve CD4^+^ transfer, which was not present in mesenteric lymph nodes (Supplementary Figure S4C,D). However, similar to our findings in chronic DSS colitis, deficiency of DC-specific PTPN2 had no effect on phosphorylation of signaling molecules (Supplementary Figure S5A,B). Together, these findings demonstrate that PTPN2-deficiency in DCs does not affect colitis severity in a T cell-driven model of intestinal inflammation.

### 2.7. Macrophage Depletion Results in Elevated Acute DSS Colitis in PTPN2-CD11cCre Mice

**Figure 7:**
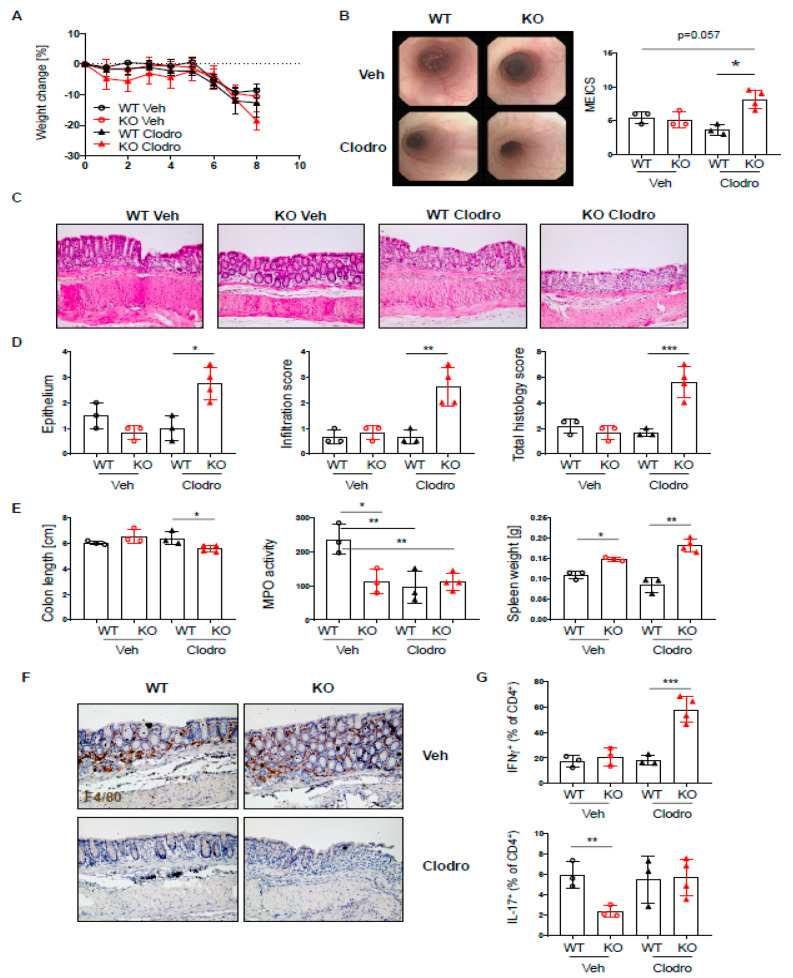
Macrophages protect PTPN2^fl/fl^xCD11c^Cre^ mice from elevated colitis. PTPN2^fl/fl^ (WT) and PTPN2^fl/fl^xCD11c^Cre^ (KO) mice received 2.5% DSS for 7 days and were treated with vehicle liposomes or clodronate liposomes on day-1, day 2, and day 4 to deplete macrophages: (**A**) weight development post injection; (**B**) representative pictures from colonoscopy and assessment of colitis severity according to the murine endoscopic index of colitis severity (MEICS); (**C**,**D**) representative pictures from H&E staining of the terminal colon and histological scoring; (**E**) colon length, MPO activity, and spleen weight; (**F**) IHC staining for F4/80^+^ macrophages in the colon; (**G**) frequencies of IFNγ^+^ and IL17^+^ CD4^+^ T cells in the lamina propria. * *p* < 0.05, ** *p* < 0.01, *** *p* < 0.001; unpaired Mann–Whitney test. Data are representative for one out of two independent experiments with 3–4 mice per experimental group.

Macrophages are important players in mucosal homeostasis, which can dictate the outcome of inflammatory reactions [29]. Since PTPN2^fl/fl^xCD11c^Cre^ mice showed elevated levels of macrophages, and these macrophages express elevated levels of PTPN2 [20], we hypothesized that macrophages might compensate for the loss of PTPN2 in dendritic cells in our mice and counteract their proinflammatory action upon colitis induction. Indeed, macrophage depletion using clodronate liposomes [30] during the induction of acute colitis resulted in elevated colitis in PTPN2^fl/fl^xCD11c^Cre^ mice but not in their WT counterparts, as observed by elevated endoscopic and histologic colitis scores (Figure 7A–C). Notably, in our setting, macrophage depletion mainly affected macrophages (CD64^+^F4/80^+^ cells), while DCs (CD64^−^/F4/80^−^/MHCII + CD11c^+^ cells) were not reduced (Supplementary Figure S6). As before, PTPN2^fl/fl^xCD11c^Cre^ mice showed reduced MPO levels (Figure 7D), and while clodronate reduced MPO in WT mice, it did not affect levels in PTPN2^fl/fl^xCD11c^Cre^ animals (Figure 7D). Clodronate efficiently depleted macrophages in WT and PTPN2^fl/fl^xCD11c^Cre^ littermates, and while levels of INF-γ^+^ and IL-17^+^ T cells were not altered in vehicle-treated PTPN2^fl/fl^xCD11c^Cre^ mice, clodronate treatment resulted in markedly elevated levels of IFN-γ^+^ T cells specifically in PTPN2^fl/fl^xCD11c^Cre^ mice. This indicates that macrophages indeed seem to counteract the proinflammatory nature of PTPN2-deficient DCs in the inflamed intestine and that their loss results in elevated colitis severity in PTPN2^fl/fl^xCD11c^Cre^ mice. 

## 3. Discussion

Here, we demonstrated that PTPN2 in DCs affects immune cell infiltrations in the lamina propria, but its presence in DCs seems to be dispensable in the context of colonic inflammation. Dysfunction of PTPN2 in DCs triggered changes in myeloid and lymphoid immune cell populations and increased infiltration of lymphocytes, macrophages, and DCs. The key role of PTPN2 in regulating immune reactions during intestinal inflammation has been described for innate and adaptive immune cells using mice deficient for PTPN2 in macrophages or T cells, which resulted in an elevated susceptibility to experimental colitis [17,19]. Together with the observation that the loss of PTPN2 in DCs affects immune cell populations, this led us to expect that PTPN2 in DCs would exert a key role in regulating immune responses during intestinal inflammation. However, in a model of acute colitis, the extent of colitis in PTPN2^fl/fl^xCD11c^Cre^ was comparable to their wild-type littermates, whereas in a model of chronic colitis, mice with PTPN2-deficient DCs tended to exhibit even less severe intestinal inflammation, while there was no effect in T cell-mediated colitis. 

DSS-induced intestinal inflammation is characterized by a dramatic erosion of the epithelial barrier, enabling commensals and pathogens to cross the epithelial barrier and trigger immune reactions [31,32]. Antigen-presenting cells (APCs) such as macrophages and DCs are important in driving and shaping the induction of immune reactions in mucosal tissues by taking up and presenting antigens. Among APCs, macrophages are the most abundant cell type in the intestinal lamina propria, essentially contributing to intestinal homeostasis by producing anti-inflammatory cytokines such as TGFβ and IL-10 and promoting function and survival of FoxP3^+^ Tregs [24,33]. Intestinal macrophages perform a central role in maintaining intestinal homeostasis by mediating wound repair and may even serve as a potential target to control intestinal inflammation [34]. Our results indicated that in a steady state, the loss of PTPN2 in DCs altered infiltration of various immune cells in the lamina propria, with remarkable infiltrations of macrophages, monocytes, and DCs. Our experiments with macrophage depletion show that the increase of PTPN2-expressing macrophages in the lamina propria upon loss of PTPN2 in DCs can explain why we did not observe spontaneous inflammation or differences regarding the severity of intestinal inflammation upon colitis induction. It is well possible that upon erosion of the epithelial barrier following DSS exposure or upon colitis induction via transfer of CD4^+^ naïve T cells, PTPN2-expressing macrophages are able to take up antigens and trigger subsequent anti-inflammatory immune reactions in PTPN2^fl/fl^xCD11c^Cre^ mice. There is also evidence that in the setting of DSS colitis, intestinal DCs regulate intestinal homeostasis via mediating the trafficking of monocytes to the site of inflammation and restraining the inflammatory function of macrophages [23]. In line with this, we show that macrophages and other phagocytes were able to compensate for the loss of PTPN2 in DCs, likely by restraining the inflammation and establishing intestinal homeostasis to a similar extent as in wild-type mice in the context of intestinal inflammation. We have addressed the important anti-inflammatory macrophages-specific function of PTPN2 in previous studies, showing elevated intestinal inflammation in mice lacking PTPN2 in macrophages upon DSS exposure [19]. Given the increased expression of PTPN2 in macrophages of PTPN2^fl/fl^xCD11c^Cre^ mice [20], it is not surprising that those macrophages exhibit increased anti-inflammatory functions and are therefore able to compensate for the loss of PTPN2 in DCs. 

The precise mechanism of how DSS induces colitis is still not clear. Besides epithelial erosion, direct effects on immune cells, and especially on intestinal phagocytes that can potentially take up DSS molecules, and effects of DSS on the microbial community are plausible effects. It has been reported that under highly alkaline and highly acidic condition, sulfate might dissociate from DSS [35] and in this way could serve as a substrate for sulfate metabolizing bacteria, such as hydrogen sulfide producing species, which have been suggested to promote intestinal pathologies under certain circumstances [36]. However, it is not clear whether this mechanism plays a role in DSS-induce colitis since the pH range in the colon (around pH 5.2) [37] is not acidic enough to result in significant sulfate dissociation from DSS, and supplementation of sulfate does not induce colitis in vivo [38]. Given the profound alterations in intestinal macrophage and DC proportions in PTPN2^fl/fl^xCD11c^Cre^ mice and the important role of these cells to control potential pathobionts, alterations in the gut microbial community and potential changes in the abundance of sulfite producers might also contribute to the observed effects. Thus, it might be of interest to investigate the intestinal microbiome in these mice in follow-up experiments. 

The role of PTPN2 in IFNγ-STAT1 signaling and T cell differentiation has been addressed in previous studies [16,17]. Our data demonstrate that upon induction of intestinal inflammation, expression of Th1-related transcription factors and cytokines was increased and phosphorylation levels of STAT1 were elevated in mice lacking PTPN2 in DCs. Further, we found that the transfer of naïve CD4^+^ T cells into PTPN2^fl/fl^xCD11c^Cre^xRAG^−/−^ mice promoted the expansion of IFNγ^+^CD4^+^ T cells. However, despite an increased Th1 response upon DC-specific loss of PTPN2, we did not observe any differences in terms of colitis severity in our mouse models. 

When investigating the role of PTPN2 in DCs we found that PTPN2-deficiency results in the development of spontaneous inflammation, particularly in skin and liver, due to increased infiltration of inflammatory cells and IFNγ-producing effector T cells [20]. However, in the intestinal lamina propria, we observed an increased expression of the Treg-transcription factor FoxP3 during acute intestinal inflammation and expansion of FoxP3^+^ Tregs in the T cell transfer model. This increase in Tregs might compensate for the loss of the PTPN2-mediated anti-inflammatory responses. There is evidence that the interaction of Tregs and macrophages via IL-10 modulates immune responses and contributes to maintaining tissue tolerance [39], again indicating that macrophages are able to compensate for the potential proinflammatory effect of PTPN2 loss in DCs in the intestine. In contrast to skin and liver, where macrophages are not altered, we observed increased levels of macrophages in the colon of PTPN2^fl/fl^xCD11c^Cre^ mice, as well as slightly increased mRNA levels of *IL-10* in acute DSS and T cell transfer colitis. Furthermore, these cells expressed high levels of PTPN2, possibly due to a compensatory effect in response to an altered immune landscape. In line with this important role of macrophages to compensate for inflammatory cues of PTPN2-deficient DCs in the intestine, macrophage depletion abrogated the protection of PTPN2-CD11cCre mice from elevated intestinal inflammation. This demonstrates that under inflammatory conditions in the colon, macrophages are able to compensate for the loss of PTPN2 in DCs by exerting an inflammation-regulating function and inducing anti-inflammatory responses, hereby suppressing exaggerated inflammatory responses mediated by PTPN2 loss in DCs.

In conclusion, PTPN2 in DCs controls and regulates the immune cell composition in the intestine. DC-specific PTPN2 deficiency results in an increased abundance of innate and adaptive immune cells and the recruitment of macrophages. During induced intestinal inflammation, the loss of PTPN2 in DCs has no appreciable impact on colitis severity since those macrophages are able to compensate for the proinflammatory effect of PTPN2-deficient DCs. In summary, our data suggest that in the setting of intestinal inflammation, PTPN2 function in DCs is dispensable and can be compensated by other immune cells, and especially by macrophages, which express increased levels of PTPN2 upon DC-specific PTPN2 deletion and monocytes. 

## 4. Methods

### 4.1. Mice, Colitis Induction, and Macrophage Depletion

For the experiments, 20–25 g female littermates were used, and all mice were kept in a specific pathogen-free (SPF) facility in individually ventilated cages with food and water ad libitum. CD11c^Cre-eGFP^ mice, which express Cre recombinase under the control of the CD11c promoter, were purchased from Jackson; RAG2^−/−^ mice, which lack mature B and T cells from Janvier, and mice with PTPN2 allele 3 flanked by loxP sequences (PTPN2^fl/fl^ mice) were obtained from EUCOMM. To generate mice lacking PTPN2 specifically in DCs (PTPN2^fl/fl^xCD11c^Cre^ mice), PTPN2^fl/fl^ mice were crossed with CD11c^Cre-eGFP^ mice. PTPN2^fl/fl^xCD11c^Cre^ mice were crossed with RAG2^−/−^ mice to obtain PTPN2^fl/fl^xCD11c^Cre^xRAG^−/−^ mice. The CD11c^+^ DC-specific deletion of PTPN2 was confirmed by sorting of different immune cell subsets (B cells, T cells, Macrophages, and DCs) and subsequent qPCR for PTPN2 [20].

Acute dextran sulfate sodium (DSS) colitis was induced in age- and weight-matched females by administration of 2.5% DSS (35–50 kDa; MP Biomedicals, Irvine, CA, USA) in the drinking water for 7 days. On day 8, mice were subjected to colonoscopy and subsequently euthanized for organ collection. Chronic colitis was induced by administration of 4 cycles consisting of 7 days 1.5% DSS in the drinking water, followed by 10 days of normal drinking water. Mice were sacrificed 4 weeks after the last DSS cycle.

For transfer colitis, naïve T cells were isolated using the EasySep Mouse CD4^+^ T Cell Isolation Kit (STEMCELL Technologies, Vancouver, BC, Canada) and subsequent flow cytometry sorting of naïve T cells (defined as CD4^+^/CD62L^high^/CD44^low^/CD25^−^ cells), and 2.5 × 10^5^ naïve T cells injected intraperitoneal (i.p.) into PTPN2^fl/fl^xRAG2^−/−^ and PTPN2^fl/fl^xCD11c^Cre^xRAG2^−/−^ hosts. The purity of naïve T cells was >95%, as analyzed by flow cytometry.

For experiments with macrophage depletion, mice were injected i.p. with clodronate- or vehicle-loaded liposomes (100 µL/mouse, ready-to-use solution) on day 1, day 2, and day 5 of the DSS application. Macrophage depletion was verified using IHC and flow cytometry.

### 4.2. Endoscopic Assessment of Colitis Severity and Histological Score

Animals were anesthetized i.p. with 90–120 mg kg^−1^ body weight ketamine (Vétoquinol, Bern, Switzerland) and 8 mg kg^−1^ body weight Xylazine (Bayer, Lyssach, Switzerland). Colonoscopy was performed as described previously [19,40]. Recording was performed with the Karl Storz Tele Pack Pal 20,043,020 (Karl Storz Endoskope, Tuttlingen, Germany). Colonoscopy was scored using the murine endoscopic index of colitis severity (MEICS) scoring system, as described previously [40]. The following five parameters were assessed: (1) transparency of the colon, (2) changes of the vascular pattern, (3) fibrin visible, (4) granularity of the mucosal surface, and (5) stool consistency. Histological scoring for inflammatory infiltration and degree of epithelial damage/edema was performed on hematoxylin and eosin-stained sections of the most distal 1 cm of the mouse colon, as described previously [41].

### 4.3. RNA Isolation, RT-PCR, and Quantitative PCR

For RNA isolation, 0.5 cm long colon pieces were mechanically disrupted in a homogenization solution containing 1-Thioglycerol (Promega, Madison, WI, USA) in gentleMACS tissue disrupter tubes (Miltenyi Biotec, Bergisch Gladbach, Germany) according to the manufacturer’s instructions. RNA was isolated using the Maxwell RSC simplyRNA Tissue Kit (Promega) according to the manufacturer’s instructions. RNA concentration was estimated by measuring absorbance at 260 and 280 nm. Complementary DNA (cDNA) was synthesized using the high-capacity cDNA reverse transcription kit (Thermo Fisher Scientific, Waltham, MA, USA) following the manufacturer’s instructions. RT-PCR was performed using FAST qPCR Master Mix and predesigned Taqman assays (Thermo Fisher Scientific) on a QuantStudio 6 system using QuantStudio software (Thermo Fisher Scientific). Mouse *Actb* was used as endogenous control, measurements were performed in triplicate, and relative expression levels were calculated according to the ΔΔCT method. 

### 4.4. Protein Isolation and Western Blotting

For protein isolation, 0.5 cm long pieces were lysed in M-PER lysis buffer (Thermo Fisher Scientific), equal amounts of protein mixed with NUPAGE^®^ 4x LDS Sample Buffer (Thermo Fisher Scientific), and samples boiled for 5 min at 95 °C. Proteins were separated using SDS-polyacrylamide gel electrophoresis and transferred onto nitrocellulose membranes. Membranes were blocked with blocking solution (3% milk, 1% BSA), and primary antibody diluted in blocking solution was added and incubated overnight. After washing with TBS-T (20 mM Tris pH 7.5, 150 mM NaCl, 0.1% Tween 20), HRP-labelled secondary antibodies in blocking solution were added for 30 min. After washing with TBS-T, immunoreactive proteins were detected using an enhanced chemiluminescence detection kit (Thermo Fisher Scientific), and densitometric analysis was performed using ImageJ software. The following antibodies were used for Western blotting: phospho-STAT1 (Tyr701) (Cell Signaling, Danvers, MA, USA, #9167S), total STAT1 (Cell Signaling, #9172S), phospho-STAT3 (Tyr705) (Cell Signaling, #9131S), total STAT3 (Cell Signaling, #9139S), phospho-P38 (Thr180/Tyr182) (Cell Signaling, #9216S), total P38 (Cell Signaling, #9212S), phospho-ERK (Thr202/Tyr204) (Cell Signaling, #9101S), total ERK (Cell Signaling, #9102S), phospho-JNK (Thr183/Tyr185) (Cell Signaling, #9251S), total JNK (Cell Signaling, #9252S), anti-rabbit HRP secondary antibody (Cell Signaling, #7074V), anti-mouse HRP secondary antibody (Cell Signaling, #7076S). 

### 4.5. Myeloperoxidase (MPO) Activity Assay

Colon specimens were homogenized in 50 mM phosphate buffer (pH 6.0) and 0.5% hexadecyltrimethylammonium bromide (Sigma-Aldrich, Buchs, Switzerland) using a gentleMACS tissue homogenizer (Miltenyi). After three freeze and thaw cycles, the supernatant was mixed with 0.02% dianisidine (Sigma-Aldrich) in 50 mM phosphate buffer, pH 6.0, and 0.0005% H_2_O_2_ (Sigma-Aldrich). Myeloperoxidase activity, expressed as arbitrary units, was calculated as mean absorbance (460 nm) per incubation time (in min).

### 4.6. Immunohistochemistry

Immunohistochemistry was performed on formalin-fixed, paraffin-embedded tissue specimens. Briefly, 5 μm tissue sections were deparaffinized in Histoclear (Brunschwig, Basel, Switzerland) and rehydrated in descending concentrations of ethanol. Antigen retrieval was performed using citrate buffer, pH 6.0 (DAKO, Glostrup, Denmark) for 30 min at 98 °C. Endogenous peroxidases were blocked by incubation with 0.9% hydrogen peroxide for 15 min at room temperature (RT), blocking was performed using 3% bovine serum albumin for 1 h at RT in a wet chamber. Samples were stained for 1 h at RT with primary antibody F4/80 (clone D2S9R, Cell Signaling, #70076S), and for 1 h at RT with HRP-labeled secondary antibody. Antibody binding was visualized using a liquid DAB+ substrate chromogen system (DAKO). Then, samples were counterstained with hematoxylin and dehydrated in ascending ethanol solution and Histoclear.

### 4.7. Isolation of Lamina Propria Lymphocytes

The whole colon was opened longitudinally, rinsed with PBS, cut into 2 mm^2^ pieces, and washed with 2% FCS HBSS. The supernatant was discarded, and tissue pieces were incubated in HBSS–EDTA solution (HBSS, 2mM EDTA) at 37 °C on a shaker (300 rpm) for 15 min. Tubes were shaken vigorously, supernatant discarded, and samples were washed with HBBS and incubated in HBBS–EDTA solution at 37 °C rpm on a shaker (300 rpm) for 30 min. Tubes were again shaken vigorously, supernatant discarded, and samples were washed with HBSS. Samples were incubated in digestion solution containing Dispase (0.6 mg/mL, Gibco) and Collagenase IV (0.4 mg/mL; from Clostridium histolyticum, Sigma-Aldrich) at 37 °C on a shaker (300 rpm) for 20 min. The samples were homogenized using an 18 + 1.5-gauge needle and the homogenate was filtered through a 40 μm cell strainer. The samples were then stained with flow cytometry antibodies.

### 4.8. Flow Cytometry

Cells were incubated with antibodies in PBS for 30 min at 4 °C, followed by washing with PBS. For intracellular labeling, cells were fixed and permeabilized with BD Cytofix/Cytoperm^TM^ (BD Biosciences, San Jose, CA, USA) for 20 min at 4 °C, washed with Perm buffer (BD Biosciences), and intracellular staining was performed in Perm buffer for 30 min at 4 °C, followed by a final wash with Perm buffer. For intranuclear staining, cells were fixed and permeabilized with Transcription Factor Staining Buffer (eBioscience, San Diego, CA, USA). For intracellular cytokine staining, cells were incubated for 4 h at 37 °C in RPMI containing 10% FCS with PMA (50 ng/mL, Sigma-Aldrich), Ionomycin (1 μg/mL, Sigma-Aldrich) and Brefeldin A (1 μg/mL, Sigma-Aldrich). Samples were resuspended in PBS and analyzed on an LSR II Fortessa (equipped with 405 nm, 488 nm, 561 nm, and 640 nm laser lines; BD Biosciences) or a BD FACSymphony (equipped with 355 nm, 405 nm, 488 nm, 561 nm, and 639 nm laser lines) with FACS Diva Software. Before data acquisition, PMT voltages were adjusted manually to reduce fluorescence spillover, and single-stain controls were acquired for compensation matrix calculation. The following anti-mouse antibodies were used: CD62L-BUV737 (clone MEL-14, BD Biosciences, #565213), CD11b-BUV661 (clone M1/70, BD Biosciences, #565080), Ly6G-BUV563 (clone 1A8, BD Biosciences, #560757), CD4-BUV563 (clone GK1.5, BD Biosciences, #565709), CD24-BUV496 (clone M1/69, BD Biosciences, #564664), CD45-BUV395 (clone 30-F11, BD Biosciences, #564279), CD3-BV785 (clone 17A2, BioLegend, San Diego, CA, #100232), NK1.1-BV711 (clone PK136, BioLegend, #108745), CD4-BV711 (clone GK1.5, BD Biosciences, #563050), TNF-BV650 (clone MP6-XT22, BioLegend, #506333), CD4-BV650 (clone RM4-5, BioLegend, #100546), Ly6C-BV605 (clone HK1.4, BioLegend, #128035), CD25-BV510 (clone PC61, BioLegend, #102041), CD45-PB (clone 30-F11, BioLegend, #103126), FoxP3-PB (clone MF14, BioLegend, #126410), CD44-BV421 (clone IM7. BioLegend, #103040), CD8-PerCP-Cy5.5 (clone 53-6.7, eBioscience, #45-0081-82), IL22-PerCP-Cy5.5 (clone Poly5164, BioLegend, #516411), IFNg-PE-Cy7 (clone XMG1.2, eBioscience, #25-7311-82), CD11c-PE-Cy7 (clone N418, Thermo Fisher Scientific, #25-0114-81), B220-PE-Cy5 (clone RA3-6B2, Thermo Fisher Scientific, #15-0452-83), F4/80-PE-Cy5 (clone BM8, BioLegend, #123112), CD8-PE-CF594 (clone 53-6.7, BD Biosciences, #562283), CD64-PE (clone X54-5/7.1, BioLegend, #139304), IL13-PE (clone eBio13A, Thermo Fisher Scientific, #12-7133-82), Zombie NIR^TM^ Fiaxable Viability Kit (BioLegend, #423106), MHCII-AF700 (clone M5/114.15.2, BioLegend, #107622), CD19-APC (clone 1D3/CD19, BioLegend, #152410), IL17-APC (clone TC11-18H10.1, BioLegend, #506916). Data analysis was performed using FlowJo 10.0.x (BD Biosciences). Populations of interest were manually pregated in FlowJo software with applied compensation correction. Equal numbers of randomly selected cells from each group were then combined to visualize data using the t-distributed stochastic neighbor embedding (t-SNE) algorithm. 

### 4.9. Cell Sorting

Cells were sorted using a FACSAria III (BD Biosciences) (equipped with 405 nm, 488 nm, 561 nm, and 633 nm lasers) and a 70 μm nozzle using a four-way purity mask. Post-sort purity was >95%. Naïve CD4^+^ T cells were sorted based on surface marker expression (CD4^+^/CD62L^high^/CD44^low^/CD25^−^).

### 4.10. Statistical Analysis

All statistical analyses were carried out using GraphPad Prism v.8 (GraphPad Software). Unless otherwise stated, data are presented as means ± standard deviation (SD). Statistical significances were determined using Mann–Whitney test. A *p* value < 0.05 was considered statistically significant.

## Data Availability

All data underlying this article are available in the article itself or in its supplementary material. Study material will be made available upon request to the corresponding author.

## Supplementary Materials

The following are available online at https://www.mdpi.com/article/10.3390/ijms22136820/s1.

## Abbreviations

DC: dendritic cell; ERK: extracellular signal-regulated kinase; IBD: inflammatory bowel disease; IFN-γ: interferon γ; IL: interleukin; JNK: c-Jun N-terminal kinase; MAPK: mitogen-activated protein kinase; PTPN2: protein tyrosine phosphatase nonreceptor type 2; STAT: Signal transducer and activator of transcription; WT: wild type.
