# Supplementary material for: Macrophages Compensate for Loss of Protein Tyrosine Phosphatase N2 in Dendritic Cells to Protect from Elevated Colitis
Source: Int J Mol Sci. 2021 Jun 25;22(13):6820. doi: 10.3390/ijms22136820 (PMC8269284; doi:10.3390/ijms22136820)
Supplement: Supplementary file 1 [file ijms-22-06820-s001.zip › ijms-1246091-supplementary.pdf]

Supplementary Figure S1

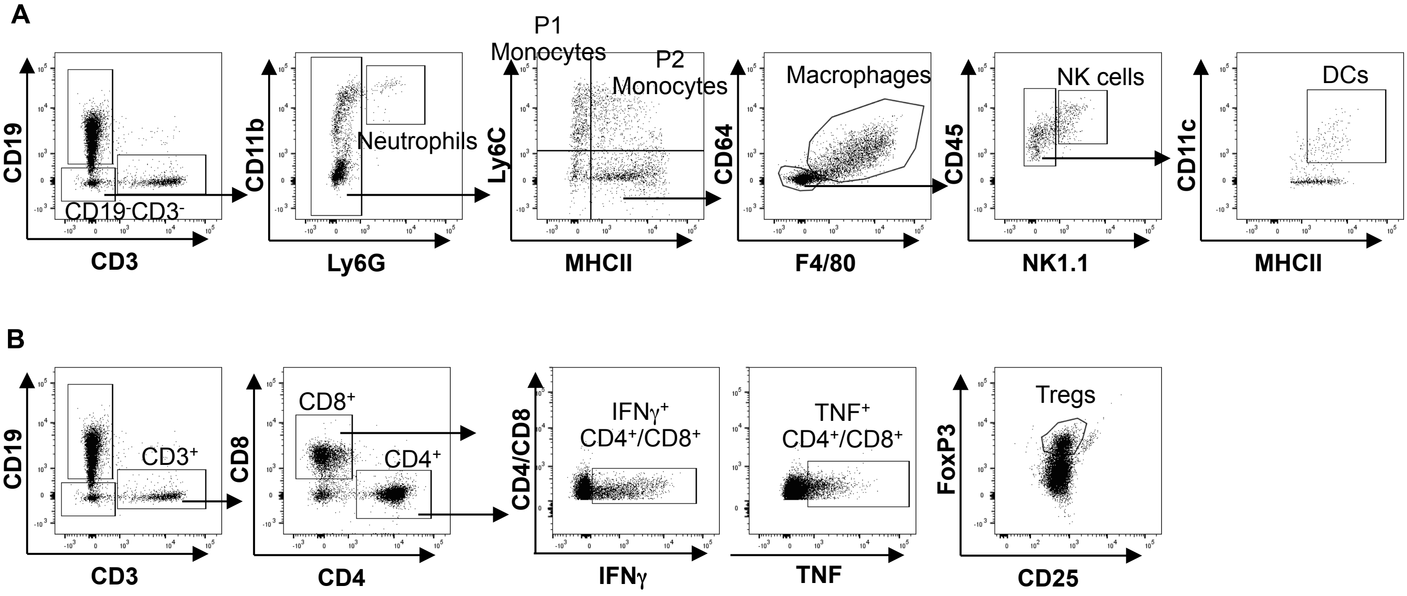

Supplementary Figure S1: Identification of major leukocyte populations based on surface marker expression. (**A**) Manual gating of flow cytometry data on major leukocyte populations. (**B**) Manual gating strategy of flow cytometry data on Tregs and cytokine-producing $CD4^+$ or $CD8^+$ T cells.

Supplementary Figure 2

Acute Colitis

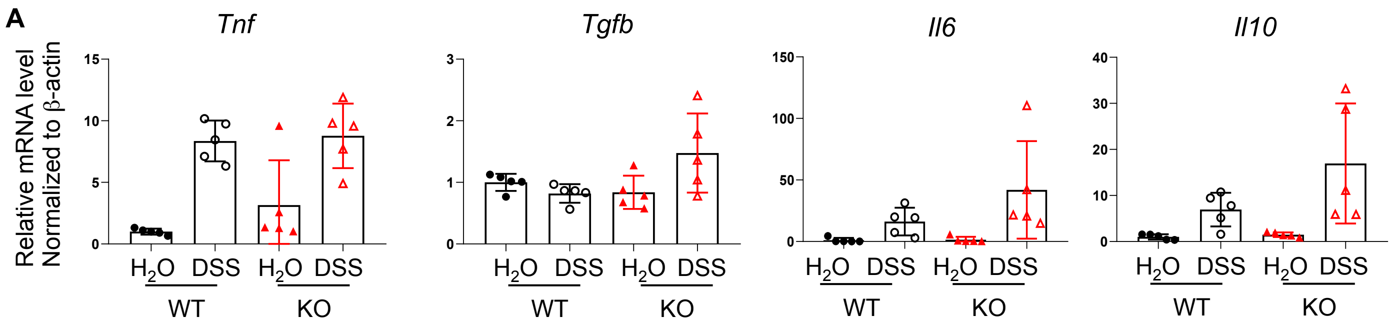

Chronic Colitis

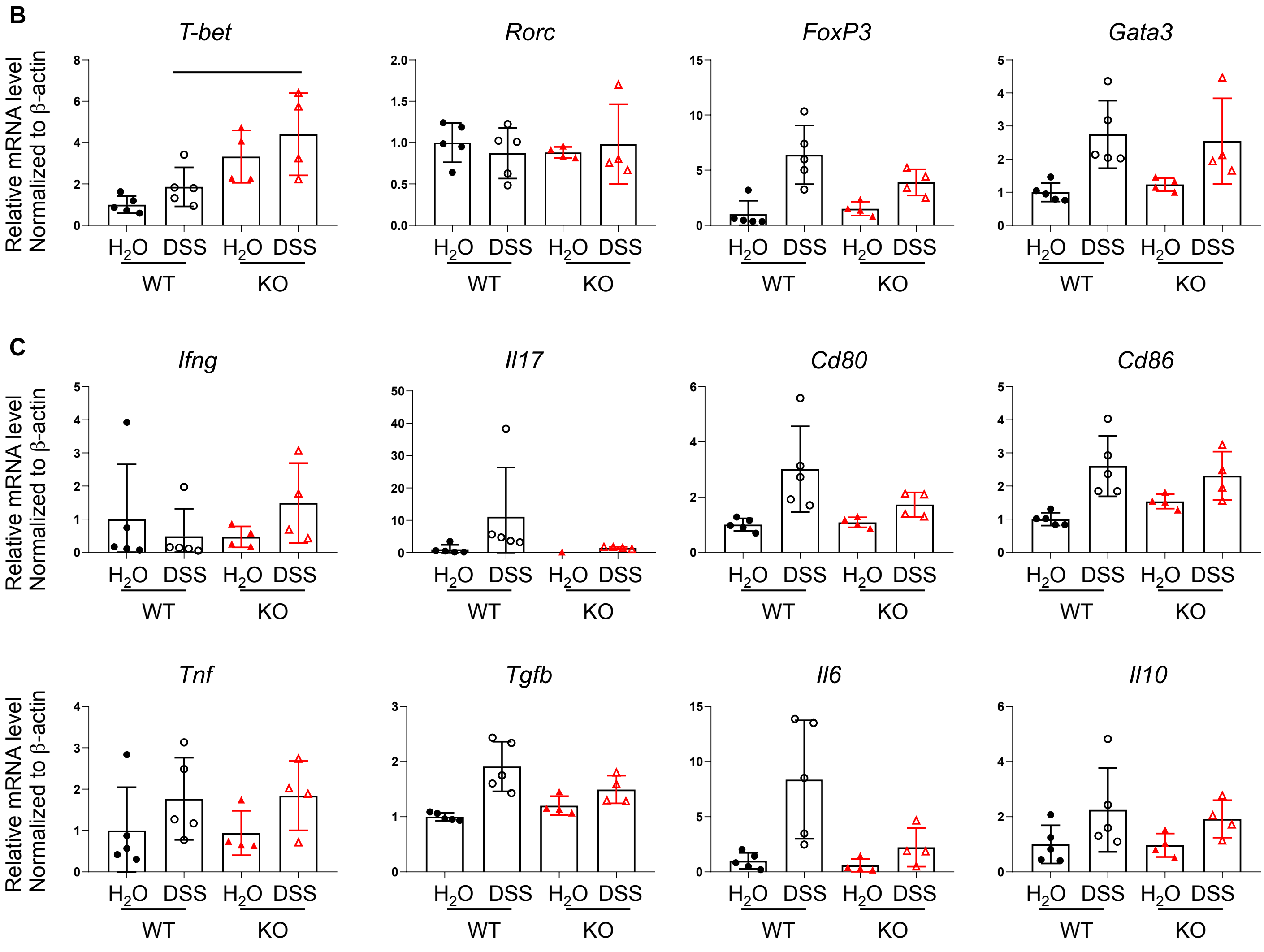

Supplementary Figure 2: Loss of PTPN2 in DCs has no effect on transcription factor and cytokine expression in chronic colitis. RNA was isolated from whole colon pieces of PTPN2$^{fl/fl}$ (WT) and PTPN2$^{fl/fl}$xCD11c^{Cre} (KO) mice. (A-C) mRNA expression of the indicated (A) cytokines in acute colitis, (B) Th-cell-associated transcription factors and (C) cytokines and activation markers in chronic colitis. *P<0.05; unpaired Mann Whitney test. Data is representative for one out of two independent experiments with 5 mice per experimental group.

Supplementary Figure 3

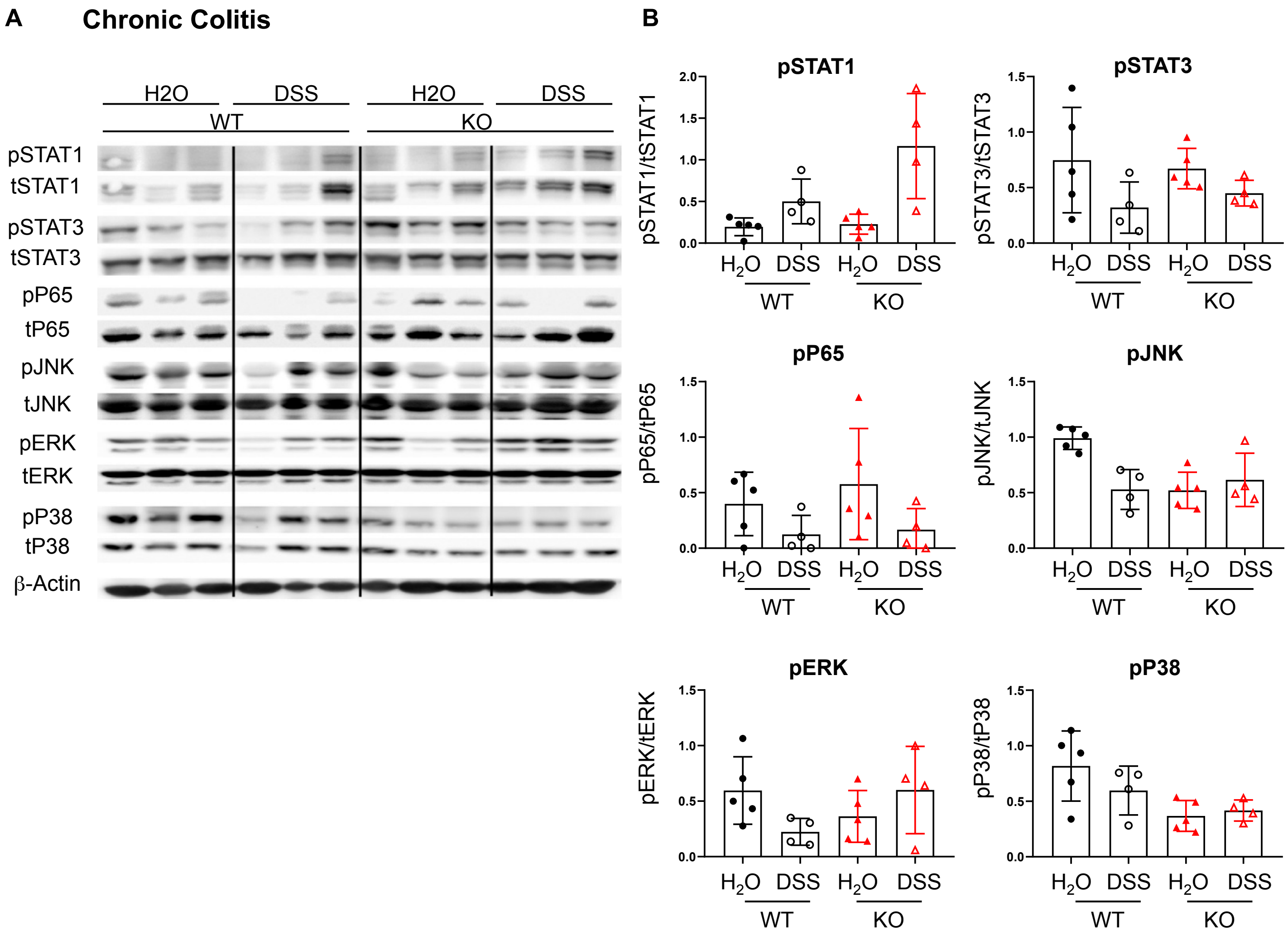

Supplementary Figure 3: DC-intrinsic PTPN2 does not regulate phosphorylation levels of PTPN2 targets in chronic colitis. Protein lysates from whole colon pieces of PTPN2$^{fl/fl}$ (WT) and PTPN2$^{fl/fl}$xCD11c^{Cre} (KO) mice were analyzed by Western blot for the indicated proteins. (A+B) Phosphorylation levels of PTPN2 targets in chronic DSS model. Data is representative for one out of two independent experiments with 5 mice per experimental group.

Supplementary Figure 4

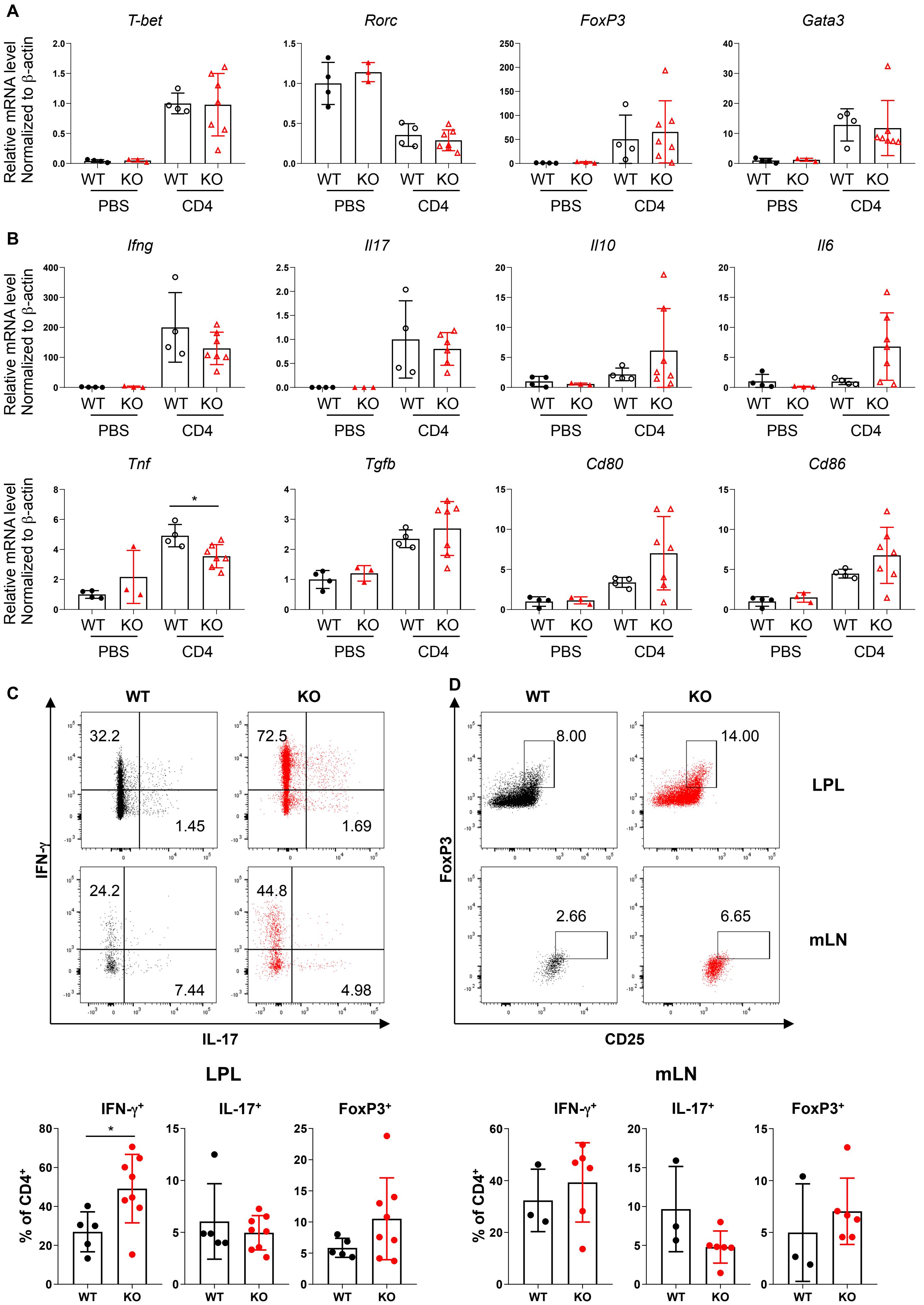

Supplementary Figure 4: Increased Th1 response and Treg induction in PTPN2$^{fl/fl}$xCD11c^{Cre}xRAG$^{-/-}$ mice in T cell transfer colitis model. PTPN2$^{fl/fl}$xRAG$^{-/-}$ (WT) and PTPN2$^{fl/fl}$xCD11c^{Cre}xRAG$^{-/-}$ (KO) mice were injected i.p. with PBS or 2.5x10^{5} naive CD4^{+} T cells. (A and B) mRNA expression of the indicated (A) Th-cell-associated transcription factors, (B) cytokines and activation markers in colon lysates. (C+D) Representative flow cytometry dot plots from lymphocytes isolated from LPL and mLN and stained for (C) IL-17 and IFNg or (D) CD25 and FoxP3 expression, gated on CD4^{+} T cells. *$P<0.05$; unpaired Mann Whitney test. Data is representative for one out of two independent experiments with 3-5 mice per experimental group.

Supplementary Figure 5

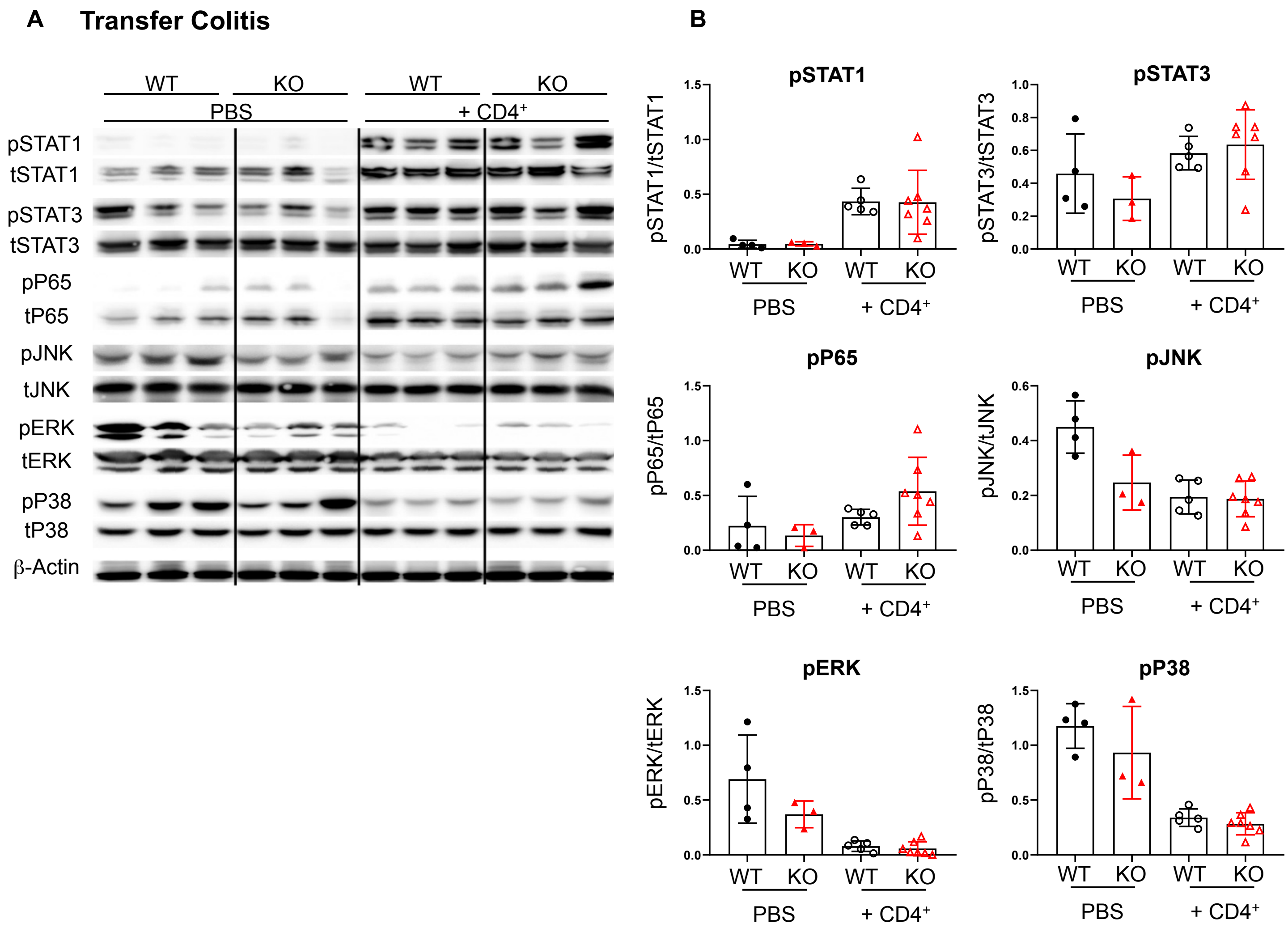

Supplementary Figure 5: DC-intrinsic PTPN2 does not regulate phosphorylation levels of PTPN2 targets in T-cell transfer colitis. Protein lysates from whole colon pieces of PTPN2$^{fl/fl}$xRAG$^{-/-}$ (WT) and PTPN2$^{fl/fl}$xCD11c^{Cre}xRAG$^{-/-}$ (KO) mice were analyzed by Western blot for the indicated proteins. (A+B) Phosphorylation levels of PTPN2 targets in T-cell transfer colitis model.

Supplementary Figure 6

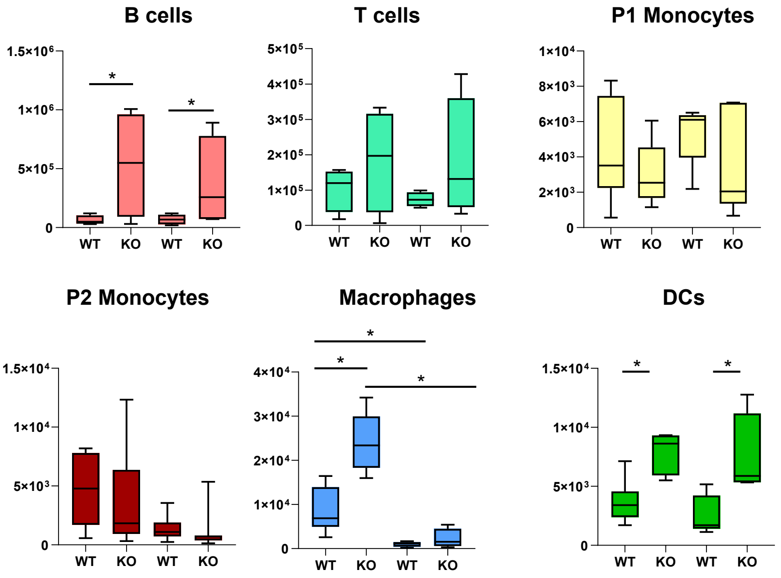

Supplementary Figure 6: Clodronate mainly depletes macrophages. PTPN2$^{fl/fl}$ (WT) and PTPN2$^{fl/fl}$xCD11c^{Cre} (KO) mice received 2.5% DSS for 7 days and were treated with vehicle liposomes or clodronate liposomes on day -1, day 2, and day 4 to deplete macrophages. Depicted are total counts of indicated cell pupulations in the lamina propria. Data is representative for one out of two independent experiments with 3-4 mice per experimental group.
